# Fate of the Arterial Origin of Major Aortopulmonary Collateral Arteries After Unifocalization

**DOI:** 10.1177/2150135120976135

**Published:** 2021-03-08

**Authors:** P.C. van de Woestijne, J.A.A.E. Cuypers, W.A. Helbing, A.J.J.C. Bogers

**Affiliations:** 1 Department of Cardio-thoracic Surgery, 6993 Erasmus University Medical Center, Rotterdam, the Netherlands; 2 Department of Adult Congenital Cardiology, 6993 Erasmus University Medical Center, Rotterdam, the Netherlands; 3 Department of Pediatric Cardiology, 6993 Erasmus University Medical Center, Rotterdam, the Netherlands

**Keywords:** aorta, pulmonary atresia, ventricular septal defect, collateral arteries

## Abstract

**Background:**

During unifocalization procedures for pulmonary atresia with ventricular septal defect and major aortopulmonary collateral arteries, collateral arteries are either ligated or detached. Not much is known of the fate of the remaining arterial origins in the long term. Available computed tomography (CT) or magnetic resonance (MR) imaging of the intrathoracic arteries was examined to investigate possible abnormalities at the former position of the collateral arteries as well as ascending aortic diameters.

**Methods:**

From 1989 to 2018, we performed 66 unifocalization procedures in 39 patients. One hundred and twenty-nine collateral arteries were ligated or detached. In 52% (15) of the surviving patients (with a total of 55 ligated or detached collaterals), sufficient imaging of the thoracic aorta from CT (11) and/or MR (9) was available for evaluation.

**Results:**

The median interval between unifocalization procedure and imaging was 15 years (interquartile range [IQR]: 9-19 years). In 93% (14) of the scanned patients, 18 blunt ends were detected at the location of a former collateral artery. No aneurysm formation of the descending aorta was observed. The median diameter of the ascending aorta was 35 mm (IQR: 31-40 mm). During follow-up, no aortic dissection or rupture occurred.

**Conclusions:**

Aortic imaging late after unifocalization showed abnormalities in 93% of the scanned patients. Abnormalities consisted mostly of blunt ends of the former collateral artery. We recommend to include routine imaging of the aorta during late follow-up to detect eventual future abnormalities and monitor aortic diameters. Ascending aortic diameters showed slight dilatation with no clinical implications so far.

## Introduction

In patients with pulmonary atresia (PA), ventricular septal defect (VSD), and major aortopulmonary collateral arteries (MAPCAs), collateral arteries to the pulmonary circulation originate from different parts of the thoracic aorta and subclavian arteries. Several surgical strategies have been described with a wide variety of handling of the MAPCAs.^1,2^ In case of dual blood supply of the lungs with intrapulmonary connections, ligation or coil closure is proposed. In patients in whom parts of the blood supply of the lungs are dependent of the MAPCAs, unifocalization procedures are performed either in a staged approach or during intracardiac correction.^1,3^ In our center, a staged approach has been followed since 1989 for all consecutive patients with acceptable and comparable results. It has been demonstrated that the histology of MAPCAs differs from aortic tissue and that MAPCAs have the tendency to become stenotic or aneurysmatic.^4^ Theoretically, the blunt ends of the ligated MAPCAs on the aortic side could dilate because of their tissue characteristics. This could lead to focal aneurysms or even aortic dissections or ruptures. Searching the literature, we found no data providing information in this regard nor did we experience this in our own patient series. In order to prevent any unexpected complication such as aortic dissection or rupture of an aneurysmatic part while follow-up length increases, we decided to review systematically our patient group. And as we analyze our results of the staged protocol in detail, we also want to address this issue. Therefore, we analyzed all available computed tomography (CT) or magnetic resonance (MR) imaging of the thoracic aorta and its branches in patients operated for PA, VSD, and MAPCAs and report our results in this article.

## Material and Methods

**Figure 1.**
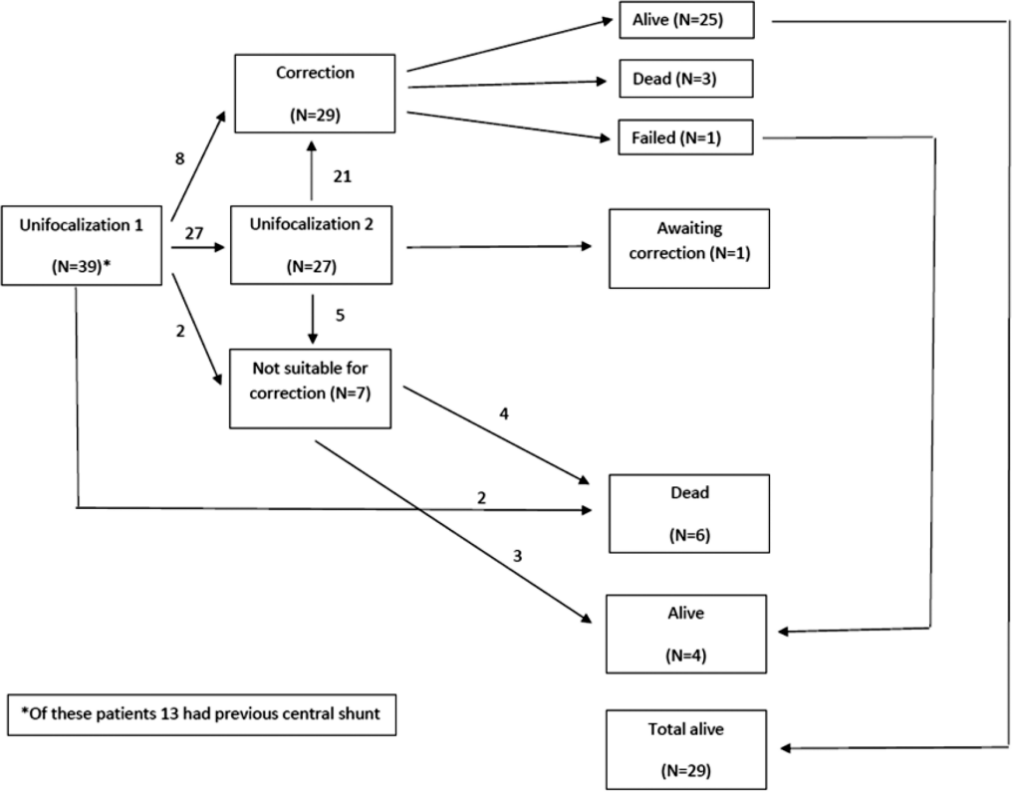
Flowchart of all consecutive patients with pulmonary atresia (PA), ventricular septal defect (VSD), and aortopulmonary collateral arteries (MAPCAs) entering our staged protocol of unifocalization and subsequent intracardiac correction.

Since 1989, we treated 39 consecutive patients with PA, VSD, and MAPCAs with our staged protocol of unifocalization and subsequent correction (see Figure 1 for patient flowchart). During 66 unifocalization procedures, 129 MAPCAs were ligated or detached from the aorta. In our patient series, no MAPCAs did arise from the subclavian arteries. The median age at first unifocalization was 13 months. Normally, we perform this procedure within four to six months of age, but some older patients treated otherwise entered our protocol when we started. For several reasons, CT and/or MR imaging was performed during follow-up. The majority of the MR scans was made for analyzing the right ventricle (RV) and therefore less suitable for aortic pathology. Computed tomography imaging was performed routinely when redo surgery was considered. From 15 of the surviving patients, sufficient CT (11) and/or MR (9) imaging of the thoracic aorta was available. In those patients, 55 MAPCAs either were ligated or detached from the aorta. Furthermore, in two patients, a remaining collateral was closed by coil placement. Patient details and study flow are listed in Table 1. The images were assessed by two investigators to identify weak spots in the aorta, aneurysm formation, or other abnormalities and diameters of the aorta were measured. The imaging was compared to angiographic imaging before unifocalization and operation reports. Clinical status was obtained from the medical records and complete. This study was approved by the institutional Ethical Committee which waived the requirement for informed consent. Statistical analysis consisted of measurement of median, and interquartile range (IQR) was performed using SPSS.

**Table 1.** Patient Characteristics and Study Flow.^a^

Patient	Operations	Age imaging	Imaging modality	Findings	Diameter of the ascending aorta
1	CS, Unifoc R, no Corr	22	CT	Blunt end, MAPCA	41
2	Unifoc L+R, Corr	20	CT	Blunt end, MAPCA	35
3	Unifoc L+R, Corr, Coil	20	MR	Blunt end	38
4	Unifoc L+R, Corr	30	CT	Blunt end, MAPCA	38
5	Unifoc L+R, Corr	38	CT	Blunt end	51
6	Unifoc L+R, Corr	22	CT + MR	Blunt end	40
7	CS, Unifoc L+R, Corr	17	MR	No	37
8	Unifoc L+R, Corr	14	MR	Blunt end	43
9	CS, Unifoc L+R, Corr	11	CT + MR	Blunt end, MAPCA	34
10	Unifoc L+R, Corr	11	CT	Blunt end, MAPCA	24
11	Unifoc R, Corr	3	CT	Blunt end	27
12	Unifoc L+R, Corr	17	CT + MR	Blunt end	27
13	Unifoc L, Corr, Coil	17	MR	Blunt end, MAPCA	31
14	CS, Unifoc L, no Corr	23	CT + MR	Blunt end	43
15	Unifoc L+R, Corr	12	CT	Blunt end, MAPCA	32

Abbreviations: Corr, correction; CS, central shunt; CT, computed tomography; MAPCA, major aortopulmonary collateral artery; MR, magnetic resonance; Unifoc, unifocalization.

^a^ Age at imaging in years and diameter of the ascending aorta in millimeters.

## Results

During follow-up, there were no records of aortic dissection or rupture and no sudden deaths which were suspicious of underlying aortic pathology. The slide thickness with CT imaging varied from 1 to 3 mm and with MR imaging from 2 to 8 mm. Therefore, some MR imaging was not suitable for detailed assessment of the thoracic arteries. Most CT images were of good quality for assessing the aortic and subclavian arteries. The median age at unifocalization of the 15 patients with sufficient imaging was 18 months (IQR: 10-40 months). Median age at scanning was 17 years (IQR: 12-22 years). The median interval time between unifocalization and imaging was 15 years (IQR: 9-19 years). Median aortic diameter at the level of the mid ascending aorta was 35 mm (IQR: 31-40 mm). This is slightly dilated but comparable with other imaging studies in patients with forms of tetralogy of Fallot.^5-7^

**Figure 2.**
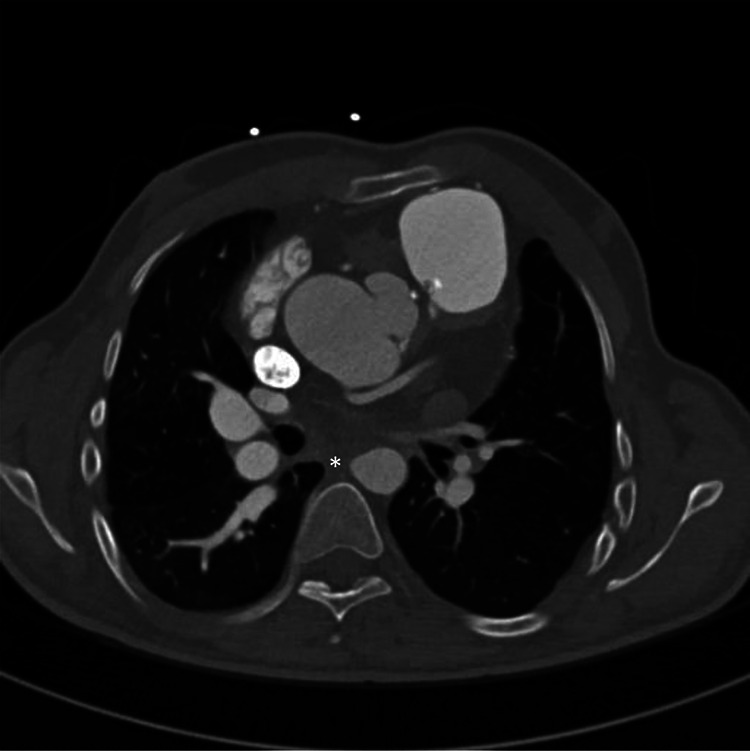
Computed tomography (CT) scan transversal image showing an oval shape of the aorta indicating a blunt end in the descending aorta (*) and an enlarged aortic root and pulmonary trunk close to the chest wall.

**Figure 3.**
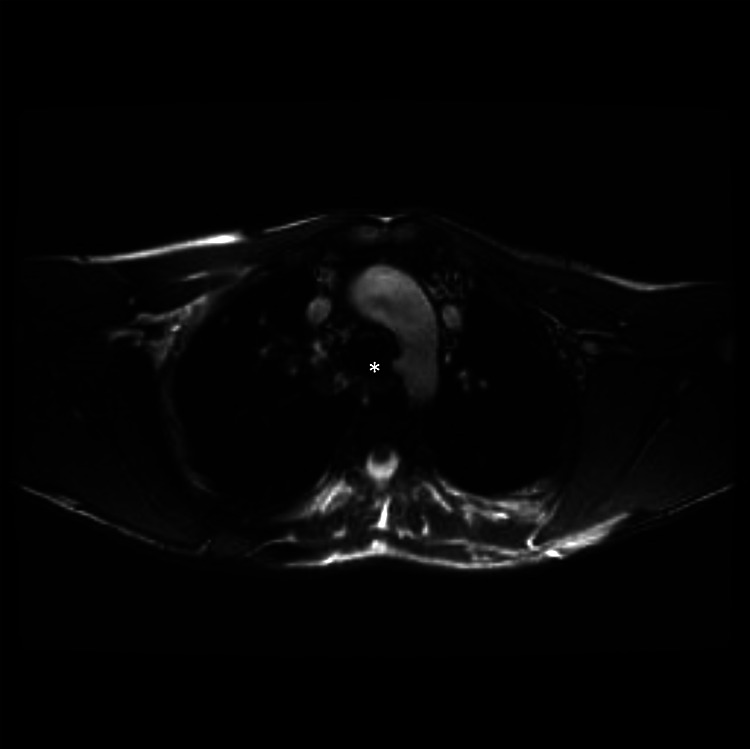
Magnetic resonance (MR) image of a blunt end (*) at a former spot of a major aortopulmonary collateral artery (MAPCA) from the proximal descending aorta. This spot corresponded to a ligated MAPCA based on angiography.

We found no marked or global aneurysm formation in the descending aorta, especially not at the former sites of collateral arteries. We also found no wall abnormalities in the descending aorta indicating weak spots. What we did found in 14 (93%) patients were small blunt ends in the descending aorta (Figures 2 and 3). Correlating these with our operation reports and angiographic imaging, they were at the site of former collateral arteries. In seven patients, we also found very small residual MAPCAs with no expected impact on pulmonary blood flow (Table 1).

## Comment

During unifocalization surgery for patients with PA, VSD, and MAPCAs, several collateral arteries were either detached from the descending aorta or ligated. This is the same for patients treated with a one-stage repair approach for this anomaly. Not much is known of the fate of the former spots of these collateral arteries a long time after surgery. We found in our series no evidence for aneurysm formation or weakened spots but only small blunt ends at the sites of former collaterals. We also found in a number of patients small residual MAPCAs with no clinical relevance. The diameters of the ascending aorta were comparable to patients with tetralogy of Fallot with some enlargement but no indication for redo surgery so far. Although these findings are satisfying, we recommend to include routine scanning of the aorta during long-term follow-up until adulthood and complete outgrowth of the aorta for this group of complex congenital patients. This could be combined for example with scanning evaluation of the right ventricular function. Special request should be made for the aorta in this regard so optimal settings are being used to obtain detailed information about aortic wall abnormalities and diameters. One consideration we could not answer is whether there is any difference in the amount of remnants of the collateral arteries in regard to the changing surgery for unifocalization from lateral thoracotomies toward median sternotomy. This could be a subject to further studies with comparison of both strategies. Limitations of this study are the small sample size and the fact that a majority of CT and MR scans were made for other reasons than detailed imaging of the aorta and may therefore be less useful. This was more pronounced when MR imaging was done for RV evaluation without detailed information about the intrathoracic arteries.

## Abbreviations

CT: computed tomography
IQR: interquartile range
MAPCA: major aortopulmonary collateral artery
MR: magnetic resonance
PA: pulmonary atresia
RV: right ventricle
VSD: ventricular septal defect
